# Altered mRNA Editing and Expression of Ionotropic Glutamate Receptors after Kainic Acid Exposure in Cyclooxygenase-2 Deficient Mice

**DOI:** 10.1371/journal.pone.0019398

**Published:** 2011-05-12

**Authors:** Luca Caracciolo, Alessandro Barbon, Sara Palumbo, Cristina Mora, Christopher D. Toscano, Francesca Bosetti, Sergio Barlati

**Affiliations:** 1 Molecular Neuroscience Unit, Brain Physiology and Metabolism Section, National Institute on Aging, National Institutes of Health, Bethesda, Maryland, United States of America; 2 Division of Biology and Genetics, Department of Biomedical Sciences and Biotechnologies and National Institute of Neuroscience, University of Brescia, Brescia, Italy; University of Nebraska Medical Center, United States of America

## Abstract

Kainic acid (KA) binds to the AMPA/KA receptors and induces seizures that result in inflammation, oxidative damage and neuronal death. We previously showed that cyclooxygenase-2 deficient (COX-2^−/−^) mice are more vulnerable to KA-induced excitotoxicity. Here, we investigated whether the increased susceptibility of COX-2^−/−^ mice to KA is associated with altered mRNA expression and editing of glutamate receptors. The expression of AMPA GluR2, GluR3 and KA GluR6 was increased in vehicle-injected COX-2^−/−^ mice compared to wild type (WT) mice in hippocampus and cortex, whereas gene expression of NMDA receptors was decreased. KA treatment decreased the expression of AMPA, KA and NMDA receptors in the hippocampus, with a significant effect in COX-2^−/−^ mice. Furthermore, we analyzed RNA editing levels and found that the level of GluR3 R/G editing site was selectively increased in the hippocampus and decreased in the cortex in COX-2^−/−^ compared with WT mice. After KA, GluR4 R/G editing site, flip form, was increased in the hippocampus of COX-2^−/−^ mice. Treatment of WT mice with the COX-2 inhibitor celecoxib for two weeks decreased the expression of AMPA/KA and NMDAR subunits after KA, as observed in COX-2^−/−^ mice. After KA exposure, COX-2^−/−^ mice showed increased mRNA expression of markers of inflammation and oxidative stress, such as cytokines (TNF-α, IL-1β and IL-6), inducible nitric oxide synthase (iNOS), microglia (CD11b) and astrocyte (GFAP). Thus, COX-2 gene deletion can exacerbate the inflammatory response to KA. We suggest that COX-2 plays a role in attenuating glutamate excitotoxicity by modulating RNA editing of AMPA/KA and mRNA expression of all ionotropic glutamate receptor subunits and, in turn, neuronal excitability. These changes may contribute to the increased vulnerability of COX-2^−/−^ mice to KA. The overstimulation of glutamate receptors as a consequence of COX-2 gene deletion suggests a functional coupling between COX-2 and the glutamatergic system.

## Introduction

Cyclooxygenases (COX-1 and COX-2) convert arachidonic acid to bioactive prostaglandins (PG) and tromboxanes (TX), which have been implicated in important physiological functions [1], [2], as well as in the pathophysiology of several neurological and neurodegenerative diseases, such as stroke, epilepsy, and Alzheimer's disease [3]. Although COX-2 is typically inducible, in the central nervous system (CNS) both COX-1 and COX-2 are constitutively expressed and COX-2 is mainly detected in the perinuclear, dendritic and axonal domains of neurons, particularly in cortex, hippocampus, amygdala and dorsal horn of the spinal cord [4], [5].

We have previously demonstrated that COX-2 deficient (COX-2^−/−^), but not COX-1^−/−^ mice, are more susceptible to kainic-acid (KA)-induced seizure intensity and neuronal damage [6]. KA, the prototypic excitoxin, binds to the alpha-amino-propionic-acid/kainate (AMPA/KA) and N-methyl-D-aspartic acid (NMDA) receptors (AMPAR, KAR and NMDAR), which are subtypes of the ionotropic glutamate receptors (iGluRs) in the brain [7], inducing seizures that result in inflammation, oxidative damage and neuronal death. These processes have been implicated in neurological, neurodegenerative, and psychiatric diseases [6], [8], [9], [10], [11], [12], [13], [14], [15].

Activation of AMPA/KA and NMDA receptors elicits a number of cellular events, including the increase in intracellular Ca^2+^, production of ROS, and other biochemical events leading to neuronal cell death [16], [17], [18], [19], [20]. In recent years, neurodegeneration caused by systemic injection of KA has been widely used to investigate mechanisms of excitotoxicity mediated by excitatory neurotransmitter agonists and possible pharmacological neuroprotective interventions [6], [21].

AMPA/KA and NMDA glutamate receptors play a major role in excitatory synaptic transmission and plasticity. Their channel properties are largely determined by subunit composition of the tetrameric receptor, assembled from GluR1–4 AMPA, GluR5–6 KA1 and KA2, NR1 with NR2a–d or NR3a–b, receptor subunits. Molecular diversity of AMPA/KA and NMDA receptors under physiological or pathological conditions is generated by differential spatial-temporal patterns of GluR expression, by alternative RNA splicing and editing and by targeting and trafficking of receptor subunits at dendritic spines [22]. In particular, RNA editing is an important post-transcriptional event in gene modification that alters one or more translation codons, thereby giving rise to functionally distinct proteins from a single gene [23]. The editing positions have been named on the basis of amino acid substitution, such as the Q/R site in AMPA GluR2 and KA GluR5 and GluR6 [23], the R/G site in GluR2, GluR3 and GluR4 [24] and the I/V and Y/C site in GluR6 [25]. These amino acid changes lead to modification of the channel properties: Q/R site profoundly influences the ionic properties of AMPA and KA receptor [26], [27] and it may determine the maturation and cellular trafficking of GluR2 [28], [29], whereas the R/G site influences the kinetic aspect of channel gating [24], [30]. The GluR6 I/V and Y/C editing sites may be involved, together with the Q/R site, in a finer regulation of ion permeability [25]. Unlike GluR2 Q/R site, which is always fully edited, the editing levels of the other sites are developmentally [31] and regionally regulated [32], [33].

It is well known that excess release of excitatory neurotransmitters, such as glutamate and KA, is an important underlying cause of neuronal damage in cerebral ischemia, epilepsy, Parkinson's disease, and Alzheimer's disease [34], [35], [36]. This type of excitation-induced neuronal damage is frequently accompanied by excess calcium influx and followed by generation of reactive oxygen and nitrogen species, which cause damage to intracellular membranes and trigger apoptotic pathways leading to delayed cell death [21].

The aim of this study was to investigate mRNA editing regulation of AMPA/KA receptor subunits and mRNA expression of all ionotropic glutamate receptor subunits (AMPA/KA and NMDA) in the cortex and hippocampus of COX-2^−/−^ and wild type (WT, COX-2^+/+^) mice after KA exposure, and to determine whether altered editing or expression of these receptors underlies the increased susceptibility of COX-2^−/−^ mice to KA-induced seizure intensity and neuronal damage. Furthermore, we showed that pretreatment with celecoxib for 2 weeks recapitulated the effects on gene expression of some AMPA, KA and most of the NMDA receptor subunits observed in COX-2^−/−^ mice, after KA treatment.

We also investigated the neuroinflammatory response of COX-2^−/−^ and WT mice to systemic KA injection by measuring the expression of inflammatory markers in the brain. We demonstrate that COX-2^−/−^ mice show an increase in the gene expression of microglia (CD11b) and astrocyte (GFAP) markers, pro-inflammatory cytokines (TNF-α, IL-1β and IL-6), and inducible nitric oxide syntase (iNOS).

## Materials and Methods

### Ethics Statement

All animal experiments were performed under an animal protocol (NICHD #08-026) approved by the National Institutes of Health (NIH), National Institute of Child Health and Disease (NICHD) Animal Care and Use Committee, in accordance with the NIH guidelines on the care and use of laboratory animals.

### Animal housing

Six week-old male COX-2^−/−^ and WT mice on a C57Bl/6-129/Ola background were received from a private NIEHS colony maintained by Taconic Farms (Germantown, NY) separately from their commercially available colony [37], [38]. All mice used in this study were progeny derived from heterozygous by heterozygous matings and therefore all contained the same strain and genetic background. Mice were maintained on a 12 h light/dark cycle with free access to food and water intake. For celecoxib pretreatment, WT mice were given free access for two weeks to a diet containing 6000 ppm celecoxib. Celecoxib (Celebrex™) capsules (400 mg; Pfizer Inc., New York, NY) were obtained from the NIH Division of Veterinary Medicine and were incorporated into feed by Research Diets, Inc. (New Brunswick, NJ) [6].

### Kainate injection

KA injection was performed as previously described [6]. Briefly, 12–14-week-old male mice were injected intraperitoneally (i.p.) with 10 mg/kg KA (Biomol International, Plymouth Meeting, PA; 2 mg/ml in 0.9% saline) or vehicle (0.9% saline). This dose of KA caused seizures but did not result in fatalities in WT mice [6]. Mice were euthanized after 24 h after KA injection and brains used for molecular analysis were rapidly dissected, frozen in 2-methylbutane at −50°C, and stored at −80°C until use.

### Gene expression

Fresh frozen mouse hippocampus and cerebral cortex were processed for RNA extraction using the Qiagen RNeasy Lipid Tissue Mini kit (Qiagen, Valencia, CA), as directed by the manufacturer. RNA quantification and quality control were done using both spectrophotometric analysis and the AGILENT Bioanalyzer 2100 lab-on-a-chip technologies. Retro-Transcription (RT) was done using the Moloney murine leukemia virus-reverse transcriptase (MMLV-RT) (Invitrogen). 2.5 µg of total RNA were mixed with 2.2 µl of 0.2 ng/µl random hexamer (Invitrogen), 10 µl of 5× buffer (Invitrogen), 10 µl of 2 mM dNTPs, 1 µl of 1 mM DTT (Invitrogen), 0.4 µl of 33 U/µl RNasin (Promega), 2 µl MMLV-RT (200 U/µl), in a final volume of 50 µl. The reaction mix was incubated at 37°C for 2 h and then the enzyme heat inactivated at 95° for 10 min. To perform the PCR reactions different amount of the RT product were mixed with 2.5 µl 10× buffer (Polymed), 0.7 µl of 1.5 mM MgCl_2_, 2.5 µl of 2 mM dNTP, 0.7 µl of each forward and reverse primer, 1.25 U of Taq polymerase in a final volume of 25 µl [39].

Quantitative real-time polymerase chain reaction (RT-PCR) was performed on selected genes AMPA GluR1–4 (Applied Biosystems TaqMan Gene Expression Assay id probes: GluR1 Mm00433753_m1; GluR2 Mm00442822_m1; GluR3 Mm00497506_m1; GluR4 Mm00444754_m1); KA GluR5–7, KA1 and KA2 (GluR5 Mm00446882_m1; GluR6 Mm00599860_m1; GluR7 Mm01179716_m1; KA1 Mm00615472_m1; KA2 Mm00433774_m1); NMDA NR1, NR2a–d and NR3a–b (NR1 Mm00433800_m1; NR2a Mm00433802_m1; NR2b Mm00433820_m1; NR2c Mm00439180_m1; NR2d Mm00433822_m1; NR3a Mm01341723_m1; NR3b Mm00504568_m1); cytokine (TNFα: Mm00443258_m1; IL-1β: Mm00434228_m1; IL-6: Mm01210733_m1); glial fibrillary acid protein (GFAP: Mm01253033_m1); microglial marker (CD11b: Mm00434455_m1); transcription factor (NF-kB: Mm00501346_m1); microtubule associate protein 2 (Mtap2: Mm00485230_m1); Nitric oxide species (iNOS: Mm00440485_m1) and phosphoglicerate kinase 1 (pgk1: Mm01225301_m1) as reference genes. PCR reactions were performed using the Applied Biosystems 7500 system. Data were analyzed using the comparative threshold cycle (ΔΔ Ct) method [40]. Results were normalized with Pgk1 as the endogenous control, and expressed as fold difference from the vehicle-injected WT mice, as previously reported [41], [42].

### Quantification of editing levels

The editing level quantification for AMPA GluR2, GluR3 and GluR4 and KA GluR5 and GluR6 transcripts was done by RT-PCR and sequence analysis [33]. Briefly, following amplification of the region containing the editing site, a pool of GluR cDNA was obtained in which both the edited and unedited mRNA forms were co-expressed. The PCR products were sequenced and the edited nucleotide appeared as overlapping A/G peaks: A from unedited transcripts and G from the edited ones [33]. The percentage of edited mRNA molecules in a pool of specific GluR mRNAs can be determined by calculating the peak area of the edited nucleotide (G) versus the sum of G and A peak areas using the software DS gene that permit to analyze the area of the peak. We previously determined that the editing level can be reliably calculated as a function of the ratio between the G peak area and A plus G peaks areas [33]. The nucleotide areas were quantified by the Discovery Studio Gene 1.5 program (Accelrys Inc., San Diego, CA, USA) [43]. The mean values and standard errors from each group of animals were used for statistical analysis.

### Statistical analysis

Editing and expression data were obtained by amplifying the mRNA from each animal. The mean values and standard errors obtained from each group were reported. Editing data were analyzed with Student's *t*-test. For RT- PCR results, a two-way ANOVA was performed on the log-transformed ΔΔ Ct. Bonferroni's post-hoc test was used for further comparisons. p values<0.05 were considered statistically significant. Data obtained in celecoxib-treated animals were analyzed using a one-way ANOVA, followed by a Bonferroni's post-hoc test.

## Results

### mRNA expression of AMPA and KA glutamate receptor subunits is decreased in COX-2^−/−^ mice after KA injection

Using qRT-PCR, we examined the relative changes in the mRNA expression pattern of the AMPA (GluR1–4) and KA (GluR5–7, KA1 and KA2) glutamate receptors subunits in the hippocampus and cortex of vehicle-injected COX-2^−/−^ mice (n = 9) compared with vehicle-injected WT mice (n = 9). Furthermore, we analyzed the expression levels of the receptor subunits of KA-injected WT (n = 9) and COX-2^−/−^ mice (n = 9) compared with their respective controls. Also we analyzed the mRNA expression of KA-injected COX-2^−/−^ mice compared with KA-injected WT mice.

**Figure 1:**
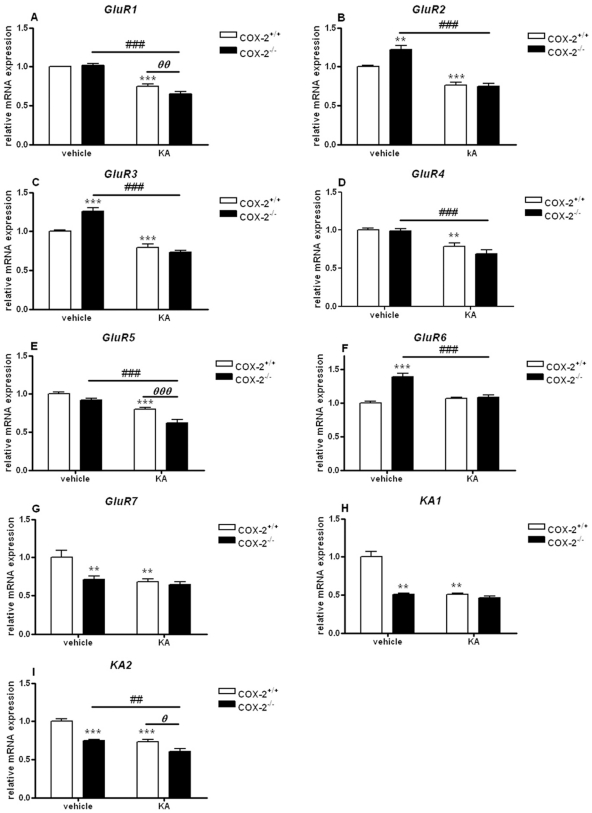
KA-induced mRNA expression of AMPA/KA receptor subunits in the hippocampus of COX-2^+/+^ and COX-2^−/−^ mice. Quantitative real time-PCR analysis of *GluR1* (A), *GluR2* (B), *GluR3* (C), *GluR4* (D) *GluR5* (E), *GluR6* (F), *GluR7* (G), *KA1* (H), *KA2* (I) for COX-2^+/+^ and COX-2^−/−^ mice 24 h after i.p. injection of KA or vehicle. Data are means ± SEM (*n* = 9). Statistical analysis was performed using a two-way ANOVA and Bonferroni's post-hoc test to compare replicate means. *P<0.05, **P<0.01, ***P<0.001 compared to vehicle-injected COX-2^+/+^ mice; #*P*<0.05, ##*P*<0.01, ###*P*<0.001, compared to vehicle-injected COX-2^−/−^ mice; ***⊖*** *P*<0.05, ***⊖⊖*** *P*<0.01, ***⊖⊖⊖*** *P*<0.001 between KA-injected COX-2^−/−^ and KA-injected WT mice.

In the hippocampus (Fig. 1A–I) of vehicle-injected COX-2^−/−^ mice the mRNA expression of GluR2, GluR3 and GluR6 was significantly increased (GluR2: 1.22±0.06, p<0.001; GluR3: 1.26±0.04, p<0.001; GluR6: 1.39±0.05, p<0.001), as compared with vehicle-injected WT mice (Fig. 1B, C; Fig. 1F). These data indicate that COX-2 deletion alters the transcription rate of glutamate receptor genes.

KA injection affected the expression pattern of several AMPA and KA receptor subunits both in WT and COX-2^−/−^ mice. Specifically, the mRNA expression of all GluR subunits (except GluR6) from KA-injected WT mice showed a decrease of about 20–50% when compared with vehicle-injected WT mice (Mean ± SEM; GluR1: 0.75±0.04, p<0.001; GluR2: 0.76±0.04, p<0.001; GluR3: 0.79±0.04, p<0.001; GluR4: 0.78±0.05, p<0.01; GluR5: 0.80±0.02, p<0.001; GluR7: 0.68±0.04, p<0.001; KA1: 0.50±0.02, p<0.001; KA2: 0.73±0.03, p<0.001) (Fig. 1A–I).

Furthermore, KA-injected COX-2^−/−^ mice showed a significant decrease in the mRNA expression of all AMPA and KA subunits (except GluR7 and KA1) (GluR1: 0.62±0.04, p<0.001: GluR2: 0.56±0.02, p<0.001; GluR3: 0.57±0.02, p<0.001; GluR4: 0.66±0.03, p<0.01; GluR5: 0.67±0.07, p<0.05; GluR6: 0.75±0.02, p<0.001; KA2: 0.8±0.06, p<0.01), as compared with vehicle-injected COX-2^−/−^ mice (Fig. 1A–I). Small but significant changes of about 20–30% were observed for the GluR1, GluR5 and KA2 subunits (GluR1: 0.82±0.06, p<0.01: GluR5: 0.72±0.05, p<0.001; KA2: 0.80±0.04, p<0.05) between KA-injected COX-2^−/−^ mice and KA-injected WT mice (Fig. 1A, 1E–1I).

**Figure 2:**
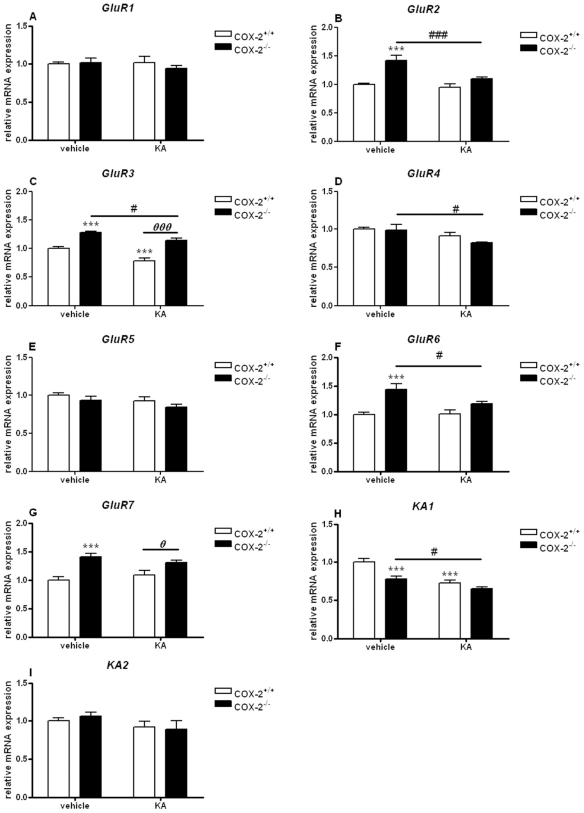
KA-induced expression of AMPA/KA receptor subunits in the cortex of COX-2^+/+^ and COX-2^−/−^ mice. Quantitative real time-PCR analysis of *GluR1* (A), *GluR2* (B), *GluR3* (C), *GluR4* (D), *GluR5* (E), *GluR6* (F), *GluR7* (G), *KA1* (H), *KA2* (I) for COX-2^+/+^ and COX-2^−/−^ mice 24 h after i.p. injection of KA or vehicle. Data are means ± SEM (*n* = 9). Statistical analysis was performed using a two-way ANOVA and Bonferroni's post-hoc test to compare replicate means. *P<0.05, **P<0.01, ***P<0.001 compared to vehicle-injected COX-2^+/+^ mice; #*P*<0.05, ##*P*<0.01, ###*P*<0.001, compared to vehicle-injected COX-2^−/−^ mice; ***⊖*** *P*<0.05, ***⊖⊖*** *P*<0.01, ***⊖⊖⊖*** *P*<0.001 between KA-injected COX-2^−/−^ and KA-injected WT mice.

In the cortex, similarly to the changes observed in the hippocampus, we found an increase in the expression of GluR2, GluR3, GluR6 and GluR7 (GluR2: 1.42±0.09, p<0.001; GluR3: 1.28±0.02, p<0.001; GluR6: 1.44±0.10, p<0.001; GluR7: 1.40±0.06, p<0.001) in vehicle-injected COX-2^−/−^ mice compared with WT mice (Fig. 2B–C, 2F–G).

Furthermore, the mRNA expression of GluR3 and KA1 subunits was decreased in KA-injected WT mice compared to vehicle-injected WT mice (GluR3: 0.78±0.05, p<0.001; KA1: 0.72±0.03, p<0.01) (Fig. 2C, 2I). Moreover, a down-regulation in the mRNA expression of AMPA GluR2–3 and KA GluR6–7 was observed in KA-injected COX-2^−/−^ mice compared with vehicle-injected COX-2^−/−^ mice (GluR2: 0.78±0.02, p<0.001; GluR3: 0.89±0.04, p<0.05; GluR6: 0.74±0.02, p<0.05; GluR7: 0.87±0.02, p<0.05) (Fig. 2B–C, 2F, 2H).

### mRNA expression of NMDA glutamate receptor subunits is decreased in COX-2^−/−^ mice after KA injection

Next, we examined the relative changes in the mRNA expression pattern of the NMDA (NR1, NR2a–d and NR3a–b) glutamate receptors subunits in the hippocampus and cortex of vehicle-injected COX-2^−/−^ mice (n = 9) compared with vehicle-injected WT mice (n = 9). Then, we analyzed the expression levels of the same receptor subunits of KA-injected WT (n = 9) and COX-2^−/−^ mice (n = 9) compared with their respectively controls. Also we analyzed the mRNA expression of KA-injected COX-2^−/−^ mice compared with KA-injected WT mice.

**Figure 3:**
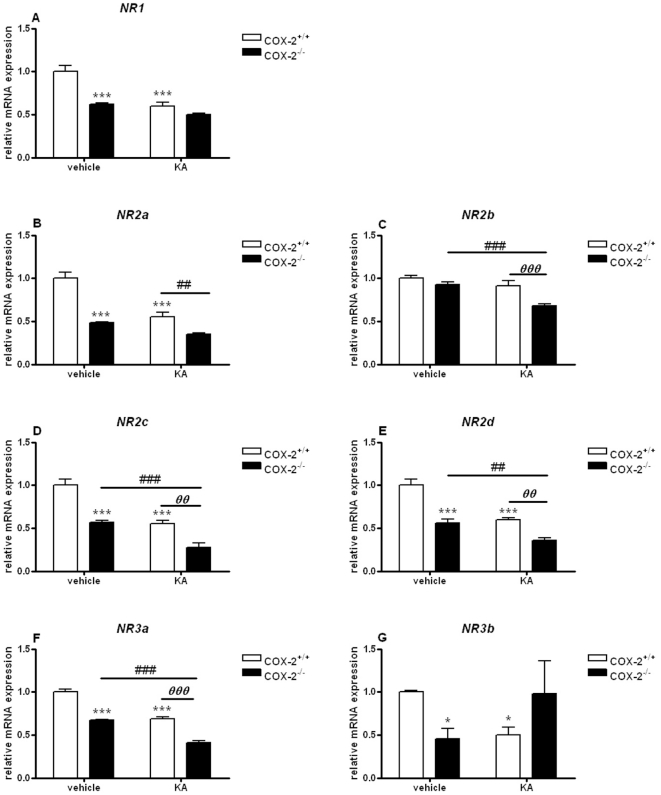
KA-induced expression of NMDA receptor subunits in the hippocampus of COX-2^+/+^ and COX-2^−/−^ mice. Quantitative real time-PCR analysis of *NR1* (A), *NR2a* (B), *NR2b* (C), *NR2c* (D), *NR2d* (E) *NR3a* (F), *NR3b* (G) for COX-2^+/+^ and COX-2^−/−^ mice 24 h after i.p. injection of KA or vehicle. Data are means ± SEM (*n* = 9). Statistical analysis was performed using a two-way ANOVA and Bonferroni's post-hoc test to compare replicate means. *P<0.05, **P<0.01, ***P<0.001 compared to vehicle-injected COX-2^+/+^ mice; #*P*<0.05, ##*P*<0.01, ###*P*<0.001, compared to vehicle-injected COX-2^−/−^ mice; ***⊖*** *P*<0.05, ***⊖⊖*** *P*<0.01, ***⊖⊖⊖*** *P*<0.001 between KA-injected COX-2^−/−^ and KA-injected WT mice.

In the hippocampus of vehicle-injected COX-2^−/−^ mice the mRNA expression of all NMDA receptor subunits, except for NR2b, was significantly decreased as compared with vehicle-injected WT mice (NR1: 0.62±0.02, p<0.001; NR2a: 0.48±0.01, p<0.001; NR2c: 0.57±0.02, p<0.001; NR2d: 0.60±0.02, p<0.001; NR3a: 0.67±0.01, p<0.001; NR3b: 0.46±0.12, p<0.05) (Fig. 3A–G). Furthermore, the mRNA expression of all NMDA subunits (except NR2b) from KA-injected WT mice were decreased when compared with vehicle-injected WT mice (NR1: 0.59±0.04, p<0.001; NR2a: 0.55±0.05, p<0.001; NR2c: 0.55±0.04, p<0.001; NR2d: 0.56±0.04, p<0.001; NR3a: 0.68±0.03, p<0.001; NR3b: 0.50±0.09, p<0.05) (Fig. 3A–G). Also, KA-injected COX-2^−/−^ mice showed a significant decrease in the mRNA expression of all NR subunits, except NR1, NR2a and NR3a, as compared with vehicle-injected COX-2^−/−^ mice (NR2b: 0.71±0.02, p<0.001: NR2c: 0.48±0.1, p<0.001; NR2d: 0.59±0.07, p<0.01; NR3a: 0.61±0.05, p<0.001) (Fig. 3A–G). A decrease of about 30–50% was observed in the expression of all NR subunits, except NR1 and NR3b, between KA-injected COX-2^−/−^ and KA-injected WT mice (NR2a: 0.63±0.03, p<0.01; NR2b: 0.70±0.02, p<0.001; NR2c: 0.49±0.14, p<0.001; NR2d: 0.60±0.07, p<0.01; NR3a: 0.60±0.05, p<0.001) (Fig. 3A–G).

**Figure 4:**
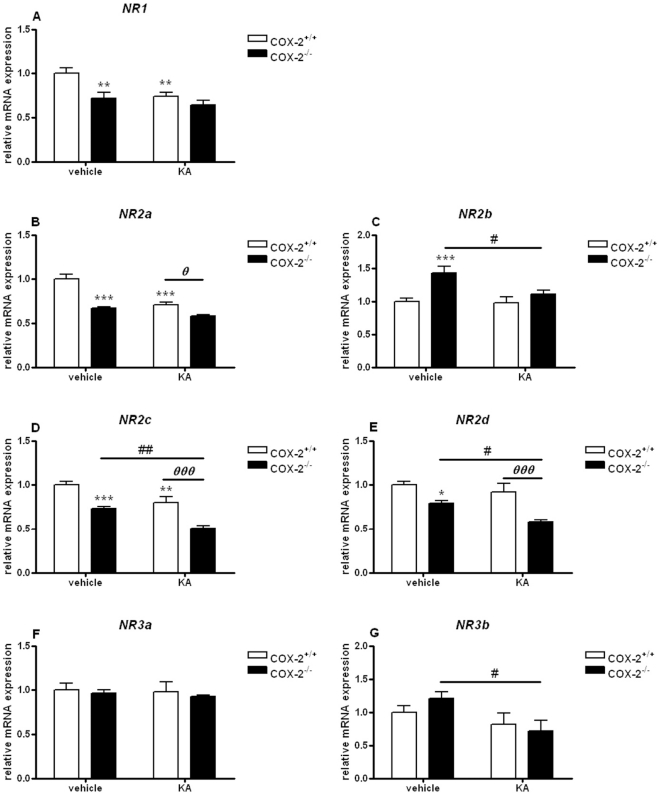
KA-induced mRNA expression of NMDA receptor subunits in the cortex of COX-2^+/+^ and COX-2^−/−^ mice. Quantitative real time-PCR analysis of *NR1* (A), *NR2a* (B), *NR2b* (C), *NR2c* (D), *NR2d* (E) *NR3a* (F), *NR3b* (G) for COX-2^+/+^ and COX-2^−/−^ mice 24 h after i.p. injection of KA or vehicle. Data are means ± SEM (*n* = 9). Statistical analysis was performed using a two-way ANOVA and Bonferroni's post-hoc test to compare replicate means. *P<0.05, **P<0.01, ***P<0.001 compared to vehicle-injected COX-2^+/+^ mice; #*P*<0.05, ##*P*<0.01, ###*P*<0.001, compared to vehicle-injected COX-2^−/−^ mice; ***⊖*** *P*<0.05, ***⊖⊖*** *P*<0.01, ***⊖⊖⊖*** *P*<0.001 between KA-injected COX-2^−/−^ and KA-injected WT mice.

In the cortex, the expression of NR1, NR2a, NR2c and NR2d subunits was decreased when we compared vehicle-injected COX-2^−/−^ (NR1: 0.72±0.06, p<0.001; NR2a: 0.67±0.02, p<0.001; NR2c: 0.73±0.02, p<0.001; NR2d: 0.79±0.03, p<0.05) with vehicle-injected WT mice (Fig. 4A–G). An increase of about 40% was observed in the expression of the NR2b subunit in vehicle-injected COX-2^−/−^ compared with vehicle-injected WT mice (NR2b: 1.42±0.09, p<0.001). Also, the expression of NR1, NR2a, NR2c and NR2d subunits was decreased when we compared KA-injected WT with vehicle-injected WT mice (NR1: 0.73±0.04, p<0.01; NR2a: 0.71±0.02, p<0.001; NR2c: 0.80±0.06, p<0.01) (Fig. 4A–G).

A down-regulation in the expression of several NR subunits (NR2b: 0.74±0.05, p<0.05; NR2c: 0.70±0.05, p<0.01; NR2d: 0.73±0.04, p<0.05; NR3b: 0. 50±0.10, p<0.05) was observed in KA-injected COX-2^−/−^ mice compared with vehicle-injected COX-2^−/−^ mice (Fig. 4A–G). A significant decrease of about 30–50% was observed in the expression of NR2a, NR2c and NR2d subunits between KA-injected COX-2^−/−^ mice and KA-injected WT mice (NR2a: 0.63±0.03, p<0.01; NR2c: 0.70±0.02, p<0.001; NR2d: 0.49±0.14, p<0.001) (Fig. 4A–G).

### Pretreatment with celecoxib altered mRNA expression of AMPA/KA and NMDA glutamate receptor subunits in WT mice after KA injection

We examined the relative changes in the mRNA expression of the AMPA (GluR1–4), KA (GluR5–7, KA1 and KA2) and NMDA (NR1, NR2a–d, NR3a–b) glutamate receptor subunits in the hippocampus and cortex of mice treated for two weeks with celecoxib, a COX-2 selective inhibitor, prior to KA injection, in vehicle-injected and KA-injected WT mice (n = 9).

**Figure 5:**
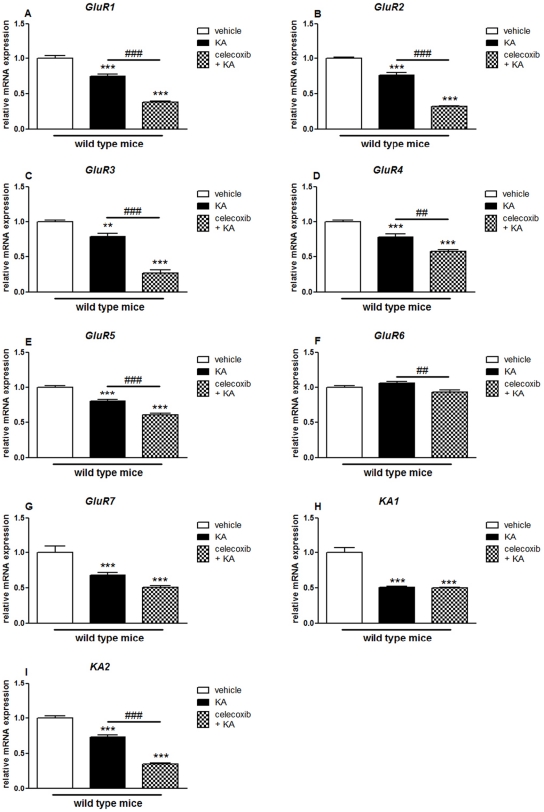
Effects of pretreatment with celecoxib on KA-induced expression of AMPA/KA receptor subunits in the hippocampus of wild type mice. Quantitative real time-PCR analysis of *GluR1* (A), *GluR2* (B), *GluR3* (C), *GluR4* (D), *GluR5* (E), *GluR6* (F), *GluR7* (G), *KA1* (H), *KA2* (I) in WT mice, after two weeks treatment with celecoxib, followed by an i.p. injection of KA or vehicle 24 hours later. Data are means ± SEM (*n* = 9). Statistical analysis was performed using a one-way ANOVA and Bonferroni's post-hoc test. *P<0.05, **P<0.01, ***P<0.001 compared to vehicle-injected WT mice; #*P*<0.05, ##*P*<0.01, ###*P*<0.001 compared to KA-injected WT mice ***⊖*** *P*<0.05, ***⊖⊖*** *P*<0.01, ***⊖⊖⊖*** *P*<0.001 between KA-injected COX-2^−/−^ and KA-injected WT mice.

**Figure 6:**
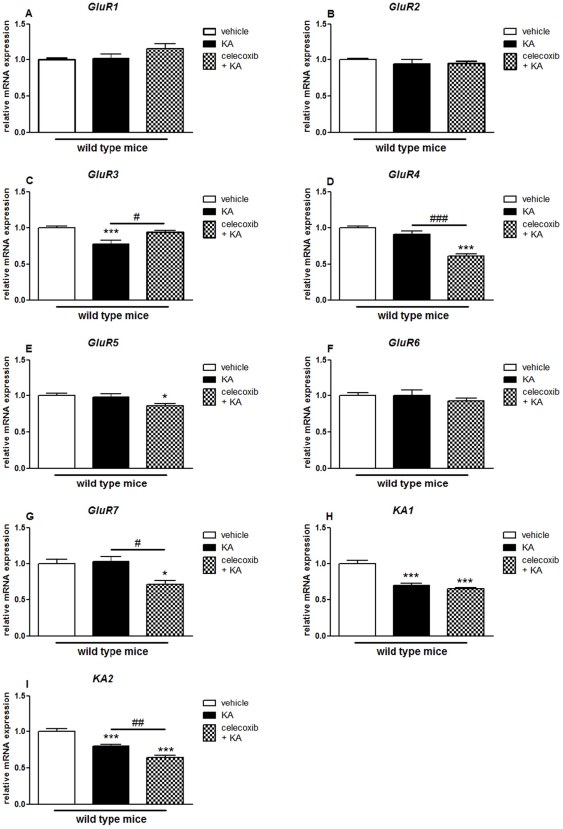
Effects of pretreatment with celecoxib on KA-induced expression of AMPA/KA receptor subunits in the cortex of wild type mice. Quantitative real time-PCR analysis of *GluR1* (A), *GluR2* (B), *GluR3* (C), *GluR4* (D) in WT mice, after two weeks treatment with celecoxib, followed by an i.p. injection of KA or vehicle 24 hours later. Data are means ± SEM (*n* = 9). Statistical analysis was performed using a one-way ANOVA and Bonferroni's post-hoc test. *P<0.05, **P<0.01, ***P<0.001 compared to vehicle-injected WT mice; #*P*<0.05, ##*P*<0.01, ###*P*<0.001 compared to KA-injected WT mice.

In the hippocampus, no changes were observed when we compared vehicle-injected and celecoxib treated mice (data not shown). KA injection affected the expression of all AMPAR, KAR (except GluR6) and NMDAR subunits in both celecoxib untreated controls (GluR1: 0.78±0.02, p<0.001; GluR2: 0.76±0.03, p<0.001; GluR3: 0.79±0.04, p<0.01; GluR4: 0.78±0.05, p<0.001; GluR5: 0.80±0.02, p<0.001; GluR7: 0.68±0.04, p<0.05; KA1: 0.50±0.02, p<0.001; KA2: 0.73±0.03, p<0.001; NR1: 0.59±0.05, p<0.001; NR2a: 0.55±0.02, p<0.001; NR2c: 0.55±0.05, p<0.001; NR2d: 0.56±0.04, p<0.001; NR3a: 0.68±0.03, p<0.001; NR3b: 0.50±0.09, p<0.001) and celecoxib-treated WT mice (GluR1: 0.38±0.02, p<0.001; GluR2: 0.32±0.01, p<0.001; GluR3: 0.27±0.01, p<0.001; GluR4: 0.57±0.02, p<0.001; GluR5: 0.60±0.02, p<0.001; GluR7: 0.50±0.02, p<0.001; KA1: 0.50±0.01, p<0.001; KA2: 0.35±0.01, p<0.001; NR1: 0.37±0.01, p<0.001; NR2a: 0.24±0.003, p<0.001; NR2b: 0.27±0.01, p<0.001; NR2c: 0.38±0.02, p<0.001; NR2d: 0.39±0.02, p<0.001; NR3a: 0.18±0.01, p<0.001; NR3b: 0.20±0.03, p<0.001) when compared with vehicle-injected WT mice (Fig. 5A–I and Fig. 6A–G).

Celecoxib pretreatment significantly decreased the mRNA expression of most iGluRs in KA-injected mice (GluR1: 0.50±0.02, p<0.001; GluR2: 0.41±0.01, p<0.001; GluR3: 0.34±0.01, p<0.001; GluR4: 0.73±0.02, p<0.01; GluR5: 0.76±0.02, p<0.001; GluR6: 0.88±0.02, p<0.01; KA2: 0.48±0.01, p<0.001; NR1: 0.62±0.02, p<0.05; NR2a: 0.43±0.01, p<0.001; NR2b: 0.30±0.01, p<0.001; NR3a: 0.27±0.02, p<0.001; NR3b: 0.41±0.05, p<0.01) (Fig. 5A–I and Fig. 6A–G).

**Figure 7:**
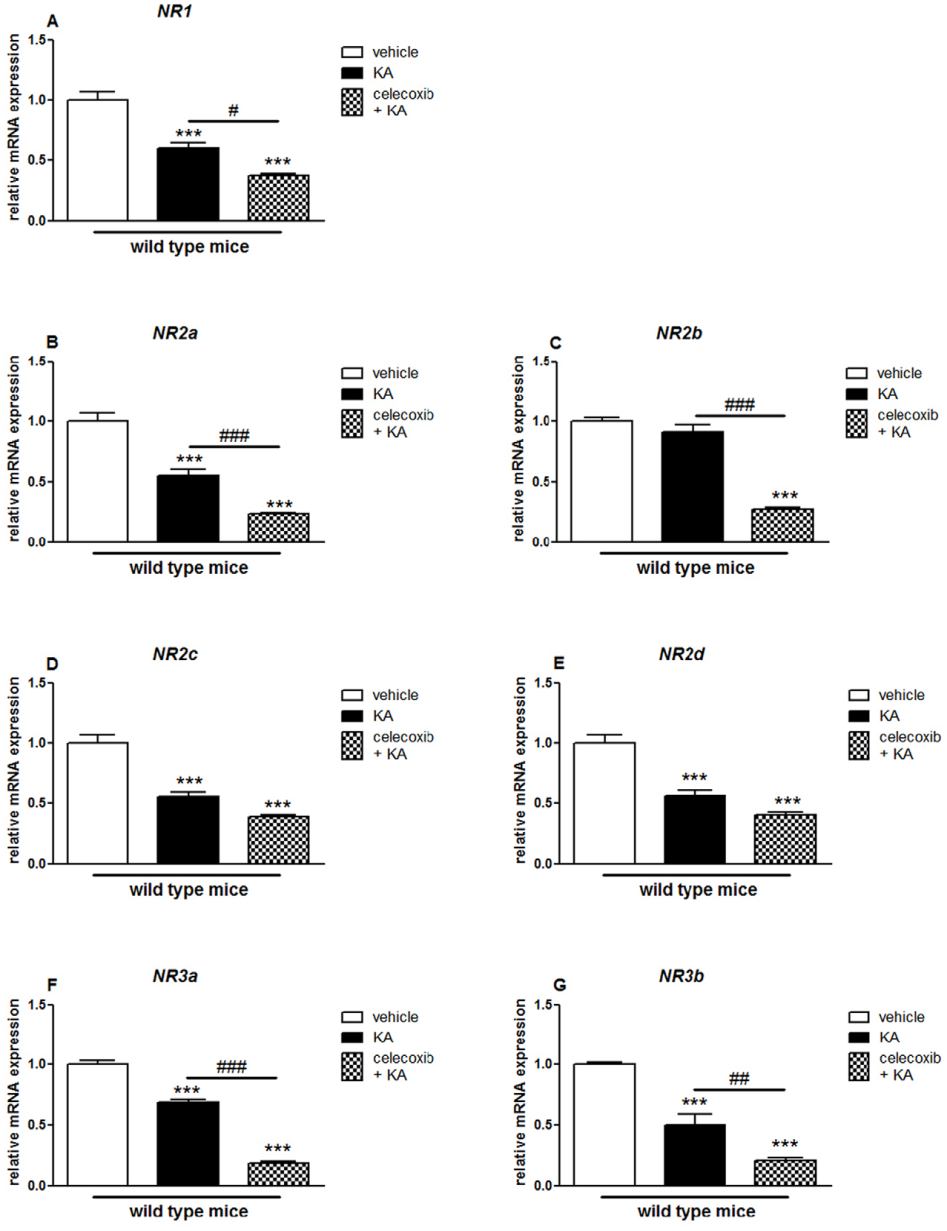
Effects of pretreatment with celecoxib on KA-induced mRNA expression of NMDA receptor subunits in the hippocampus of wild type mice. Quantitative real time-PCR analysis of *NR1* (A), *NR2a* (B), *NR2b* (C), *NR2c* (D), *NR2d* (E) *NR3a* (F), *NR3b* (G) in WT mice, after two weeks treatment with celecoxib, followed by an i.p. injection of KA or vehicle 24 hours later. Data are means ± SEM (*n* = 9). Statistical analysis was performed using a one-way ANOVA and Bonferroni's post-hoc test. *P<0.05, **P<0.01, ***P<0.001 compared to vehicle-injected WT mice; #*P*<0.05, ##*P*<0.01, ###*P*<0.001 compared to KA-injected WT mice.

**Figure 8:**
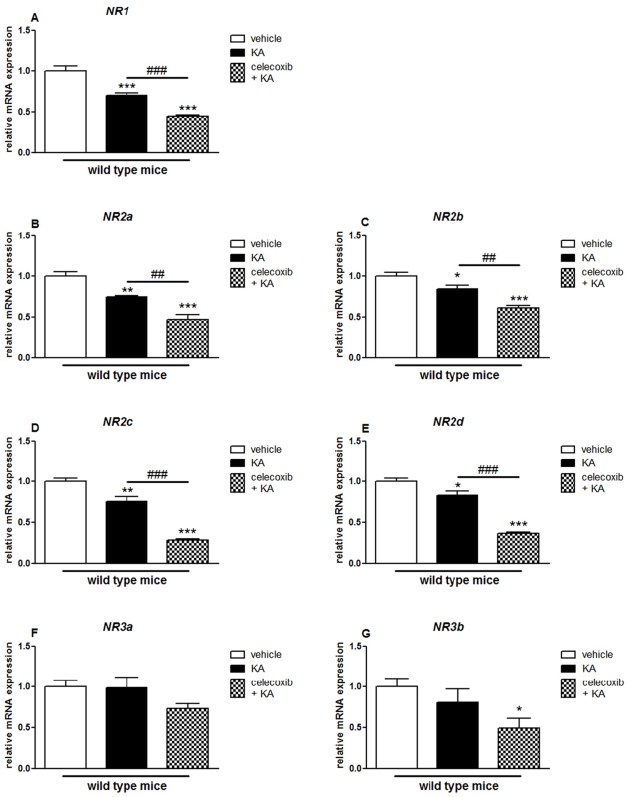
Effects of pretreatment with celecoxib on KA-induced mRNA expression of NMDA receptor subunits in the hippocampus of wild type mice. Quantitative real time-PCR analysis of *NR1* (A), *NR2a* (B), *NR2b* (C), *NR2c* (D), *NR2d* (E) *NR3a* (F), *NR3b* (G) in WT mice, after two weeks treatment with celecoxib, followed by an i.p. injection of KA or vehicle 24 hours later. Data are means ± SEM (*n* = 9). Statistical analysis was performed using a one-way ANOVA and Bonferroni's post-hoc test. *P<0.05, **P<0.01, ***P<0.001 compared to vehicle-injected WT mice; #*P*<0.05, ##*P*<0.01, ###*P*<0.001 compared to KA-injected WT mice.

In the cortex, no changes were observed when we compared vehicle-injected and celecoxib treated mice (data not shown). KA injection affected the expression of several AMPAR, KAR and NMDAR subunits in both celecoxib-untreated (GluR3: 0.78±0.05, p<0.001; KA1: 0.70±0.03, p<0.001; KA2: 0.80±0.03, p<0.001; NR1: 0.70±0.03, p<0.001; NR2a: 0.74±0.01, p<0.001; NR2b: 0.84±0.04, p<0.05; NR2c: 0.75±0.06, p<0.01; NR2d: 0.83±0.05, p<0.05) and celecoxib-pretreated mice (GluR4: 0.61±0.02, p<0.001; GluR5: 0.86±0.03, p<0.05; GluR7: 0.72±0.06, p<0.05; KA1: 0.66±0.01, p<0.001; KA2: 0.65±0.03, p<0.001; NR1: 0.45±0.03, p<0.001; NR2a: 0.47±0.06, p<0.001; NR2b: 0.61±0.03, p<0.001; NR2c: 0.29±0.01, p<0.001; NR2d: 0.37±0.01, p<0.001; NR3b: 0.49±0.12, p<0.05) when compared with vehicle-injected WT mice (Fig. 7A–I and Fig. 8A–G).

Although the effect was less robust than the one observed in the hippocampus, after KA injection celecoxib-pretreated mice showed reduced cortical mRNA expression of several iGluRs compared with celecoxib-untreated mice (GluR4: 0.67±0.03, p<0.001; GluR7: 0.69±0.06, p<0.05; KA2: 0.81±0.03, p<0.01; NR1: 0.64±0.04, p<0.001; NR2a: 0.63±0.08, p<0.01; NR2b: 0.73±0.04, p<0.01; NR2c: 0.38±0.001, p<0.001; NR2d: 0.42±0.02, p<0.001). Celecoxib pretreatement increased the expression of GluR3 (1.2±0.03, p<0.05) in response to KA injection (Fig. 7A–I and Fig. 8A–G).

### Pattern of in vivo mRNA editing levels of GluRs after KA treatment

Since the function of AMPA glutamatergic receptors is modulated by the mRNA editing of the different subunits, we analyzed editing levels of GluR2 Q/R and R/G sites, GluR3 and GluR4 R/G sites, GluR5 Q/R site and GluR6 I/V, Y/C and Q/R sites in the hippocampus and cortex. The AMPA R/G sites were analyzed in combination with the AMPA splicing variants called flip and flop [24].

In all mice tested the editing level of GluR2 Q/R site was virtually 100%, with no variations due to either KA injection or genotype (data not shown). In hippocampus and cortex, no changes were detected in the R/G site, flip- and flop-isoforms, of GluR2 in either KA- injected WT mice or in vehicle-injected COX-2^−/−^ mice when compared with vehicle-injected WT mice (Table S1, Supplementary data).

**Figure 9:**
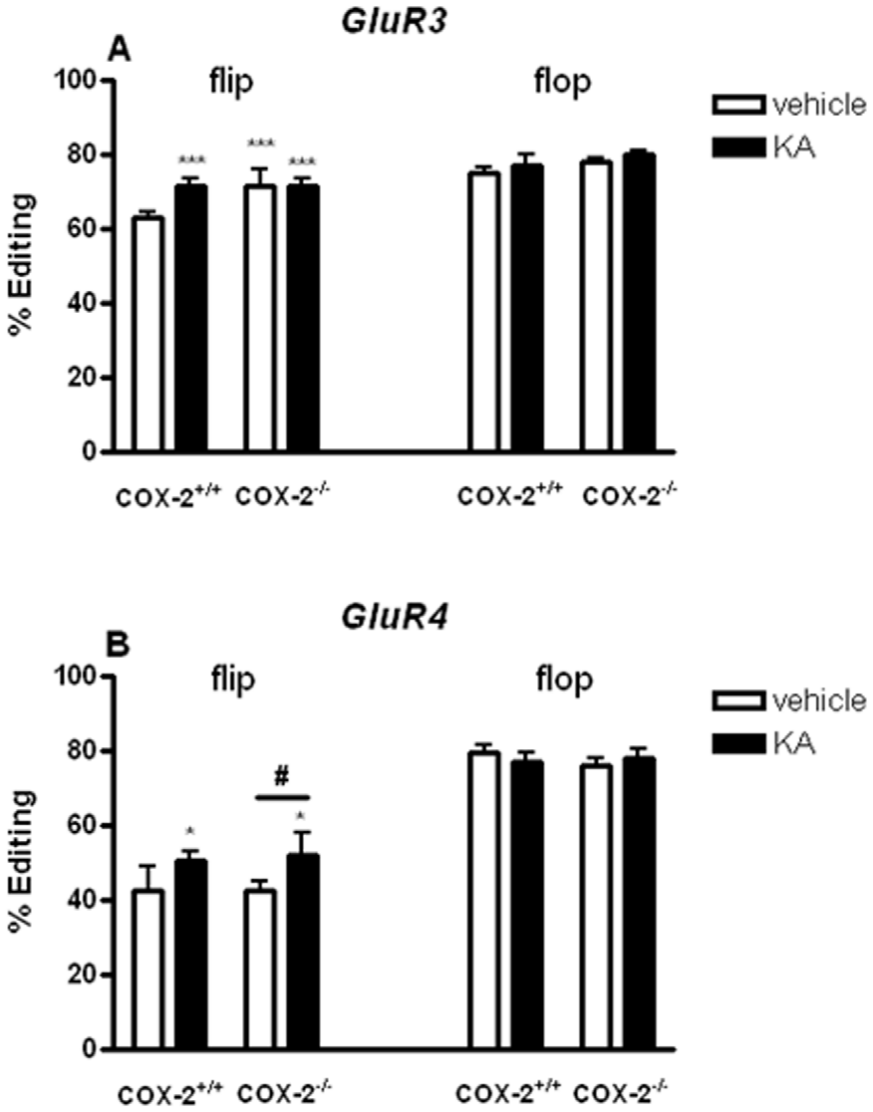
KA-induced editing of AMPA editing site glutamate receptor subunits in the hippocampus of COX-2^+/+^ and COX-2^−/−^ mice. Evaluation of RNA editing levels of the AMPA glutamate receptors in the hippocampus of mice injected with KA. (A) GluR3 R/G site flip- flop-variant and (B) GluR4 R/G flip- flop-variant, editing site. Data are presented as mean ± SEM (n = 6). Statistical analysis was performed with Student's *t* test (*P<0.05, **P<0.01, ***P<0.001).

**Figure 10:**
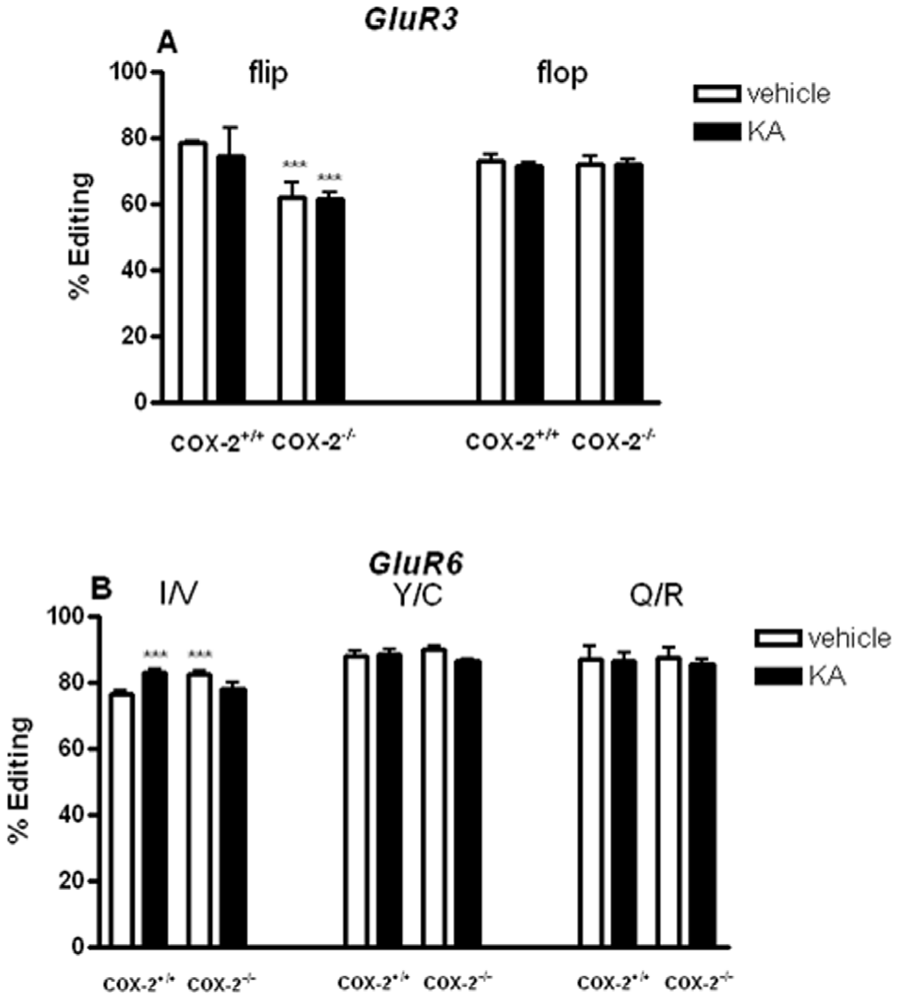
KA-induced editing of AMPA and KA glutamate receptor subunits in the cortex of COX-2^+/+^ and COX-2^−/−^ mice. RNA editing levels of AMPA and KA glutamate receptors in the cerebral cortex of mice exposed to acute KA-injection. (A) GluR3 R/G site flip- flop-variant and (B) GluR6 I/V, Y/C and Q/R editing site. Data are presented as mean ± SEM (n = 6). Statistical analysis was performed with Student's *t* test (*P<0.05, **P<0.01, ***P<0.001).

In the hippocampus, the GluR3 R/G site for the flip isoform showed a small but significant increase after KA injection in WT mice (8.4%, p<0.001) and vehicle-injected COX-2^−/−^ mice (8.4%, p<0.001) when compared to vehicle-injected WT mice (Fig. 9A). In the cortex, we found a decrease (−16.4%, p<0.001) in the GluR3 R/G site for the flip variant in vehicle-injected COX-2^−/−^ mice when compared with vehicle-injected WT mice (Fig. 10A).

Similarly, in the hippocampus, GluR4 R/G site, flip variant, showed an increase after KA injection in both WT (8%, p<0.05) and COX-2^−/−^ mice (8%, p<0.05) when compared with vehicle-injected WT mice (Fig. 9B). No changes were observed in the editing levels of GluR3 (Fig. 9A and Fig. 10A) and GluR4 R/G site (Fig. 9B and Table S1), flop-isoform, after KA injection or between genotypes in the hippocampus or cortex.

No changes by genotype or KA-treatment were found in the editing level of kainate GluR5 Q/R site in the hippocampus or in the cortex (Table S1). GluR6 Y/C and Q/R sites were not altered by KA treatment either in the hippocampus (Table S1) or cerebral cortex (Fig. 10B). However, there was a small but statistically significant increase in the I/V site in the cortex of KA-injected WT mice (p<0.001) and vehicle-injected COX-2^−/−^ mice (p<0.001) when compared with vehicle-injected WT mice (Fig. 10B). Furthermore, we analyzed editing levels of the different subunits in mice pretreated for two weeks with celecoxib. No changes due to KA-treatment or celecoxib were detected either in the hippocampus or cortex (Table S2, Supplementary data).

### mRNA expression of genes involved in the neuroinflammatory response is increased in COX-2^−/−^ mice after KA

**Figure 11:**
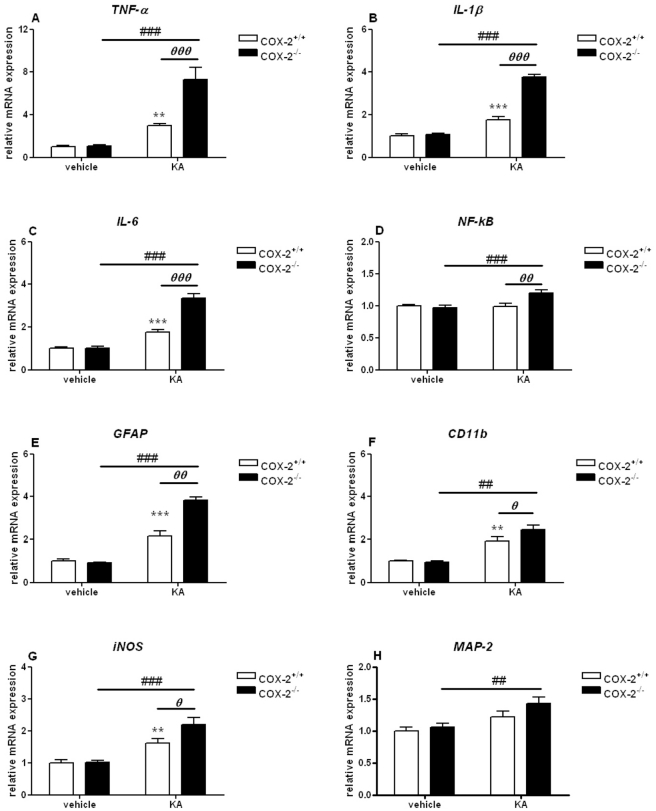
KA-induced expression of genes involved in the neuroinflammatory response in the hippocampus of COX-2^+/+^ and COX-2^−/−^ mice. Quantitative real time-PCR analysis of *TNF-α* (A), *IL-1β* (B), *IL-6* (C), *NF-kB* (D), *GFAP* (E), *CD11b* (F), *iNOS* (G), *MAP-2* (H) for COX-2^+/+^ and COX-2^−/−^ mice 24 h after i.p. injection of KA or vehicle. Data are means ± SEM (*n* = 9). Statistical analysis was performed using a two-way ANOVA and Bonferroni's post-hoc test to compare replicate means. *P<0.05, **P<0.01, ***P<0.001 compared to vehicle-injected COX-2^+/+^ mice; #*P*<0.05, ##*P*<0.01, ###*P*<0.001, compared to vehicle-injected COX-2^−/−^ mice; ***⊖*** *P*<0.05, ***⊖⊖*** *P*<0.01, ***⊖⊖⊖*** *P*<0.001 between KA-injected COX-2^−/−^ and KA-injected WT mice.

Gene expression of cytokines was determined using real time PCR in hippocampus and cortex. In the hippocampus, no changes were observed in the mRNA expression of all inflammatory markers analyzed when we compared vehicle-injected COX-2^−/−^ with vehicle-injected WT mice (Fig. 11A–C). These data indicate that COX-2 deletion *per se* does not alter the transcription rate of inflammatory markers. KA-injection significantly increased the expression of TNF-α, IL-1β and IL-6 in both COX-2^−/−^ and WT mice, however, the increase was significantly higher in the COX-2^−/−^ mice (P<0.001; Fig. 11A–C).

**Figure 12:**
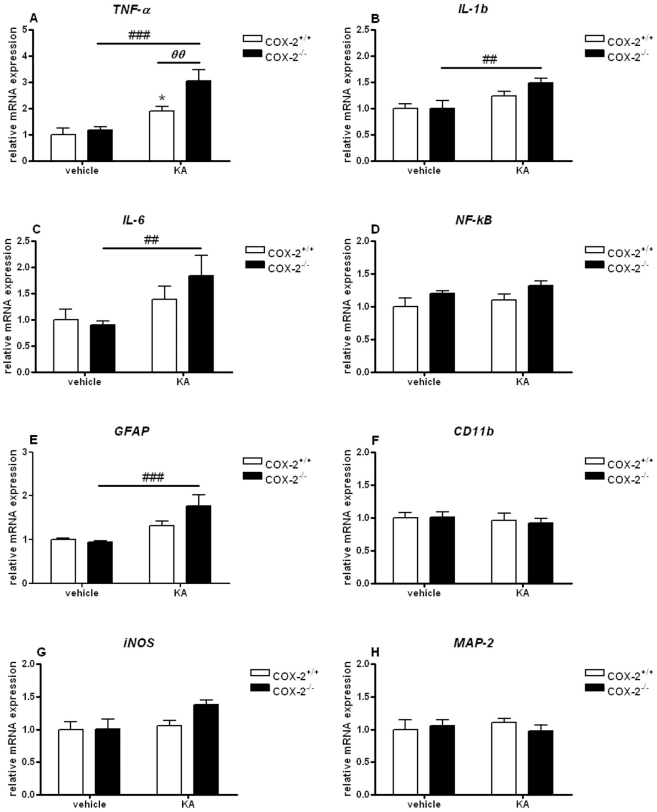
KA-induced expression of genes involved in the neuroinflammatory response in the cortex of COX-2^+/+^ and COX-2^−/−^ mice. Quantitative real time-PCR analysis of *TNF-α* (A), *IL-1β* (B), *IL-6* (C), *NF-kB* (D), *GFAP* (E), *CD11b* (F), *iNOS* (G), *MAP-2* (H) for COX-2^+/+^ and COX-2^−/−^ mice 24 h after i.p. injection of KA or vehicle. Data are means ± SEM (*n* = 9). Statistical analysis was performed using a two-way ANOVA and Bonferroni's post-hoc test to compare replicate means. *P<0.05, **P<0.01, ***P<0.001 compared to vehicle-injected COX-2^+/+^ mice; #*P*<0.05, ##*P*<0.01, ###*P*<0.001, compared to vehicle-injected COX-2^−/−^ mice; ***⊖*** *P*<0.05, ***⊖⊖*** *P*<0.01, ***⊖⊖⊖*** *P*<0.001 between KA-injected COX-2^−/−^ and KA-injected WT mice.

In the cortex, no changes was observed in the mRNA expression of TNF-α, IL-1β and IL-6 genes when we compared vehicle-injected COX-2^−/−^ with vehicle-injected WT mice (Fig. 12A–C). KA-injection increased the expression of TNF-α (3.05±0.41, p<0.001), IL-1β (1.48±0.09, p<0.01) and IL-6 genes (1.83±0.39, p<0.01) in COX-2^−/−^ mice and the expression of TNF-α (1.89±0.17, p<0.05) in WT mice (Fig. 12A–C). The increase in the gene expression of TNF-α was significantly higher in KA-injected COX-2^−/−^ than in KA-injected WT mice (1.61±0.29, p<0.05; Fig. 12A).

To determine glial cell response, we examined the gene expression of GFAP, a specific marker for astrocytes, and CD11b, a specific marker for microglia. No changes were observed in either brain areas analyzed in the mRNA expression of GFAP and CD11b when we compared vehicle-injected COX-2^−/−^ with vehicle-injected WT mice (Fig. 11E–F, Fig. 12E–F).

In the hippocampus, KA increased the expression of GFAP and CD11b, with a more significant effect in COX-2^−/−^ (GFAP: 3.83±0.16, p<0.001; CD11b: 2.46±0.20, p<0.01) than in WT mice (GFAP: 2.14±0.24, p<0.001; CD11b: 1.91±0.19, p<0.01) (Fig. 11E–F). Moreover, KA-injected COX-2^−/−^ mice showed a higher increase in the expression of GFAP (1.88±0.13, p<0.01) and CD11b (1.26±0.11, p<0.05) when compared with KA-injected WT mice (Fig. 11E–F). In the cortex, KA-injected COX-2^−/−^ showed an increase in the expression of GFAP (1.89±0.17, p<0.05) when compared to vehicle-injected COX-2^−/−^ mice. No changes were observed in the mRNA expression of CD11b (Fig. 12E–F).

No change was observed in the mRNA expression of iNOS, a major source or oxidative stress, when we compared vehicle-injected COX-2^−/−^ with vehicle-injected WT mice in the hippocampus or cortex (Fig. 11G, Fig. 12G). In the hippocampus, COX-2^−/−^ mice (2.19±0.22, p<0.001) showed a higher increase in iNOS mRNA levels in response to KA compared to WT mice (1.61±0.14, p<0.01). Also, KA-injected COX-2^−/−^ mice showed an increase in the expression of iNOS (1.36±0.15, p<0.05) when compared with KA-injected WT mice (Fig. 11G). In the cortex, no changes were observed in the expression of iNOS in any of the groups (Fig. 12G).

Furthermore, we analyzed the mRNA expression of the pro-inflammatory transcription factor NF-kB. In the hippocampus, an increase in the mRNA levels of NF-kB was observed in KA-injected compared to vehicle-injected COX-2^−/−^ mice (1.20±0.05, p<0.001) (Fig. 11D). KA-injected COX-2^−/−^ mice showed an increase in the expression of NF-kB (1.19±0.06, p<0.01) when compared with KA-injected WT mice (Fig. 11D). In the cortex, no changes were observed in the expression of NF-kB in any of the groups analyzed (Fig. 12G).

Next, we examined mRNA gene expression of MAP-2 that belongs to the microtubule-associated protein family, which is enriched in neuronal cell bodies and dendrites. In the hippocampus, KA-injection significantly increased the expression of MAP-2 (1.43±0.09, p<0.01) in COX-2^−/−^ mice (Fig. 11H). In the cortex, no change was observed in MAP-2 gene expression (Fig. 12H). The mRNA expression of COX-1 was not significantly changed in either COX-2^−/−^ and WT mice after KA exposure in either brain areas (data not shown), indicating that the increased neuroinflammatory response was not due to an increased compensatory expression of COX-1 in response to KA as a consequence of COX-2 gene deletion.

## Discussion

In this study, we report for the first time a complete analysis of mRNA expression for all subunits of AMPA (GluR1–4), KA (GluR5–7, KA1 and KA2) and NMDA (NR1, NR2a–b and NR3a–b) receptors in the hippocampus and cortex from COX-2^−/−^ and WT mice after KA injection. We used KA as a model of excitotoxicity because of its strong and well-characterized time and regional effects in inducing seizures and subsequent neuronal damage. Administration of KA has been shown to increase production of reactive oxygen species, mitochondrial dysfunction, and apoptosis in neurons in many regions of the brain, particularly in the hippocampal CA1 and CA3 subfields, and hilus of dentate gyrus. KA also produces inflammatory responses typically found in neurodegenerative diseases. Several studies suggest that KA-induced excitotoxicity can be used as a model for elucidating mechanisms underlying oxidative stress and inflammation in neurodegenerative diseases [14], [16], [44].

Our data show that AMPA GluR2–3 and KA GluR6 mRNA levels are up-regulated in the hippocampus of COX-2^−/−^ mice while the mRNA expression of all NMDA receptor subunits is down-regulated. KA injection caused a general decrease in the mRNA of AMPARs (GluR1–4), KARs (GluR5, GluR7, KA1–KA2) and NMDARs (NR1, NR2a–d, NR3a) in the hippocampus of both COX-2^+/+^ mice and COX-2^−/−^ mice, whereas in the cortex only KA1, NR1, NR2a and NR2c mRNA levels were decreased.

These data indicate that COX-2^−/−^ mice might suffer of an imbalance of glutamate receptor expression. We observed an increase in the transcription levels for AMPA (GluR2 and GluR3) and KA (GluR6) receptor subunits in both the hippocampus and cortex of vehicle-injected COX-2^−/−^ mice compared to WT mice, whereas the mRNA levels of NMDA subunits were generally decreased. The net effect of this action might be an increased glutamatergic throughput of AMPARs relative to NMDA receptors. Thus, we can speculate that genetic deletion of COX-2 alters the functional interplay between AMPA and NMDA receptors, leading to the dampening of NMDA receptors and potentiation of AMPA receptors. These data confirm a role of COX-2 in modulating the expression of glutamate receptors. Moreover, the imbalance in glutamatergic neurotransmission could contribute, at least in part, to the increased susceptibility of COX-2^−/−^ mice to KA-induced seizure intensity and neuronal damage that we previously described [6].

The NMDA receptor is formed by NR1 subunits interacting with NR2A–D, conferring functional variability depending on the specific subunits involved [45]. NR2A and NR2B are the major NR2 subunits in the adult neocortex and hippocampus. NR2A is confined to synapses of mature neurons, whereas NR2B is distributed mainly extrasynaptically [46]. Synaptic NMDA receptor activity is extremely important for neuronal survival, while the extrasynaptic NMDAR is coupled to cell-death pathways [47].

Recently, a new mechanism of autoregulation of the NMDA receptor induced by agonists overactivaction in mature neurons has been described by Gascon and colleagues [48]. The decrease in the expression of NMDAR subunits that we found in celecoxib-treated and COX-2^−/−^ mice after KA may be explained by this mechanism of autoregulation in response to glutamate activation. NMDAR overactivation may downregulate the function of synaptic receptors in neurons in response to excitotoxic neuronal degeneration. NR1 can also be regulated by a late-onset mechanism consisting of transcriptional suppression of NR1 obligatory subunit under excitotoxic conditions [49]. Inhibition of NR1 synthesis might also result in a progressive decrease in the activity of synaptic and extrasynaptic NMDARs. Calcium influx and brief stimulation of NMDARs with excitotoxic concentrations of agonist is sufficient to irreversibly reduce the level of this receptor subunit. These mechanisms of autoregulation of the NMDARs may also be involved in KA-induced changes in the expression of NMDARs and subsequent excitoxicity and neuronal degeneration.

The increased susceptibility to KA-induced excitotoxicity of COX-2^−/−^ mice raises an important issue regarding the possible involvement of COX-2 in neuroprotection and glutamatergic neurotransmission [6]. Specifically, COX-2 could play a role in attenuating glutamate excitotoxicity and, consequently, Ca^2+^ influx, by indirectly modulating the transcription of AMPA/KA and NMDA receptors.

After KA injection we observed a global down-regulation of AMPA/KA and NMDA mRNA expression, in WT and to a greater extent in COX-2^−/−^ mice. Following its depolarizing actions, KA may enhance intracellular accumulation of Ca^2+^ to promote selective neuronal damage. Neuronal cells might protect themselves from the damaging overstimulation, inducing the observed decrease in the overall glutamate receptors expression, in an attempt to maintain homeostasis.

Our data agree with a previous report by Grooms et al (2000) showing that KA administration caused delayed death of pyramidal neurons in the hippocampal CA1 and CA3 subfields that was preceded by down-regulation of GluR2 mRNA and protein expression [50]. Thus, AMPA receptor subunits may play an important role in the neurotoxicity induced by KA, although the mechanism underlying the involvement of this class of receptors in excitotoxicity should be further investigated. It is believed that the behavioral and neuropathological changes induced by KA are initiated by the activation of KA receptors in the CA3 region of the hippocampus [10], followed by release of the endogenous excitatory amino acids, glutamate, and aspartate [11], with an activation of all types of glutamate receptors.

Ohno et al (1997) [51] showed that KA-induced excitotoxicity in embryonic rat hippocampal cultures is mediated by AMPA but not KA receptors, and involves NMDA receptor-mediated toxicity through the response of KA mediated by AMPA-preferring receptors. Non-NMDA receptor-mediated excitotoxicity has been proposed to contribute to neuronal loss in a broad range of pathological conditions including hypoxia, hypoglycemia, ischemia, epilepsy, trauma [52], [53], amyotrophic lateral sclerosis [54], Huntington's disease [55], and Alzheimer's disease [56], [57].

Regulation of glutamate receptors subunits is complex and includes several intracellular steps, from transcriptional to post-translational modifications, which may have functional consequences on receptor subunit rearrangement and lead to functional differences in the functioning of synaptic circuits. Specifically, several reports clearly indicate that changes in editing of specific glutamate receptor subunits are accompanied by corresponding changes in the physiological properties of the channels [23], [26], [27], [58]. Our data indicate that KA also modulated RNA editing, a post-transcriptional mechanism known to alter functional properties of selected subunits of AMPA and KA receptors and to induce a fine-tuning modulation of glutamate neurotransmission [23]. RNA editing reaction influences the structure and, most importantly, the function of the receptor, by acting at different levels: RNA editing modulates RNA splicing of several glutamate receptors [24], and Q/R RNA editing modulates receptor transport from the endoplasmic reticulum to the plasma membrane and mediates GluR subunit tetramerization [28], [29]. At the functional level, Q/R RNA editing modulates ionic transport through GluR receptor channels [26], [27], whereas R/G RNA editing modulates desensitization and the recovery time of GluR receptors [30]. Thus, the physiological action of glutamate seems to rely greatly on a proper RNA editing reaction. Supporting this notion, recent reports show that changes in the editing level may have a profound impact on glutamatergic neurotransmission in epilepsy [59], [60], [61], amyotrophic lateral sclerosis (ALS) [62], [63], spinal cord injury [43] and malignant gliomas [64].

In our study, the GluR2 Q/R site, the most important editing site for modulation of AMPA receptor channel properties, remained fully edited after KA injection in both the brain areas analyzed, indicating that modifications in calcium permeability of AMPA receptors were not involved in KA action.

In the hippocampus, we observed an increase in GluR3 and GluR4 R/G level after KA injection in both WT and COX-2^−/−^ mice. Our data suggest that increased editing at the AMPA R/G site, by altering resensitization kinetics, could enhance the receptor response to glutamate, resulting in a synapse operating at an increased gain. The potentially higher frequency of post-synaptic receptor activation due to fast resensitization kinetics, might in this case lead to an increased Ca^2+^ concentration in the post-synaptic neuron.

On the other hand, in the cortex, we found a decrease in the GluR3 R/G site editing, flip form, in vehicle-injected COX-2^−/−^ compared with WT mice. Although no changes in editing levels were observed after KA injection, the observed decrease in GluR3 R/G site editing might cause a selective reduction of conductance for GluR3-containing receptors after KA.

Regarding the editing levels of KA receptors, KA injection selectively affected only the GluR6 I/V site in the cortex, indicating that KA slightly decreased cation permeability of the GluR6-containing receptor channels. The observed increase in I/V GluR6 levels might indicate an excitotoxin-induced decrease in the activity of voltage-gated glutamatergic channels of the KA subtype. It is possible that desensitization at KA-preferring receptors plays an important role in neuroprotection.

Furthermore, we showed that pretreatment with celecoxib for 2 weeks recapitulated the effects on gene expression of some AMPA, KA and most of the NMDA receptor subunits observed in COX-2^−/−^ mice after KA exposure, suggesting that the effects observed were not due to life-time compensatory changes that may exist in knockout mice. No alteration in the editing reaction was observed after celecoxib treatment, indicating that editing levels are extremely conserved and editing modifications may occur only after a longer treatment or in a situation of life-time inhibition of COX-2 activity as observed in COX-2^−/−^ mice.

Furthermore, we observed that COX-2 gene deletion enhanced the neuroinflammatory response to KA. KA increased mRNA expression of pro-inflammatory cytokines, such as TNF-α, IL-1β and IL-6, iNOS, a marker of oxidative stress, GFAP, a marker for astrocytes, and CD11b, a marker for microglia. These data are consistent with previous reports from our and other groups showing that COX-2 gene deletion or inhibition increases the neuroinflammatory response to endotoxins [42]. iNOS may also contribute to microglia-mediated KA induced neurotoxicity by increasing the production of extracellular reactive oxygen and nitrogen species, which, in turn, stimulate microglial release of pro-inflammatory mediators that, like radical oxygen species, are toxic to neurons.

MAP2 gene expression was increased in the hippocampus of COX-2^−/−^ mice after KA. MAP2 exhibits microtubule-stabilizing activity and regulates the microtubule networks in dendrites, resulting in dendrite elongation [65]. Thus, as reported in surviving neurons of medial extended amygdala after status epilepticus [66], KA might induce a process of sprouting and reactive synaptogenesis.

In conclusion, we have previously shown that Cox-2^−/−^ mice are more susceptible to KA-induced seizures and neuronal damage [6]. Although some of the changes observed in this model may be the consequence of adaptive modifications due to deletion of the COX-2 gene, chronic administration of the Cox-2 selective inhibitor celecoxib recapitulated these findings [6]. While we cannot directly link changes in glutamate receptor editing to susceptibility to KA, we speculate that altered mRNA editing and expression of glutamate receptors in COX-2^−/−^ mice could cause an imbalance in the interplay between AMPA and NMDA receptors and alterations in glutamatergic neurotransmission. These alterations might contribute, at least in part, to the increased susceptibility of COX-2−/− mice to KA-induced excitotoxicity [6]. Overall, our findings suggest a role of COX-2 in modulating the expression of glutamate receptors and, in turn, regulating glutamatergic neurotransmission. Future studies are warranted to elucidate the molecular mechanisms that underline the interplay between COX-2 gene and the glutamatergic system.

## Supporting Information

Table S1 **KA-induced editing of AMPA/KA glutamate receptor subunits in hippocampus and cortex of COX-2^+/+^ and COX^−/−^ mice.** Data are Means ± SEM expressed as %editing level compared to vehicle-injected COX-2^+/+^ mice. Statistical analysis was performed with Student's test.
(PPTX)

Table S2 **KA-induced editing of AMPA/KA glutamate receptor subunits in hippocampus and cortex of wild type mice after pretreatment with celecoxib.** Data are Means ± SEM expressed as % editing level compared to vehicle-injected wild type mice. Statistical analysis was performed with Student's test.
(PPTX)
