# Supplementary material for: Altered mRNA Editing and Expression of Ionotropic Glutamate Receptors after Kainic Acid Exposure in Cyclooxygenase-2 Deficient Mice
Source: PLoS One. 2011 May 12;6(5):e19398. doi: 10.1371/journal.pone.0019398 (PMC3093380; doi:10.1371/journal.pone.0019398)
Supplement: Table S1 — KA-induced editing of AMPA/KA glutamate receptor subunits in hippocampus and cortex of COX-2+/+ and COX−/− mice. Data are Means ± SEM expressed as %editing level compared to vehicle-injected COX-2+/+ mice. Statistical analysis was performed with Student's test. (PPTX) [file pone.0019398.s001.pptx]

## Slide 1
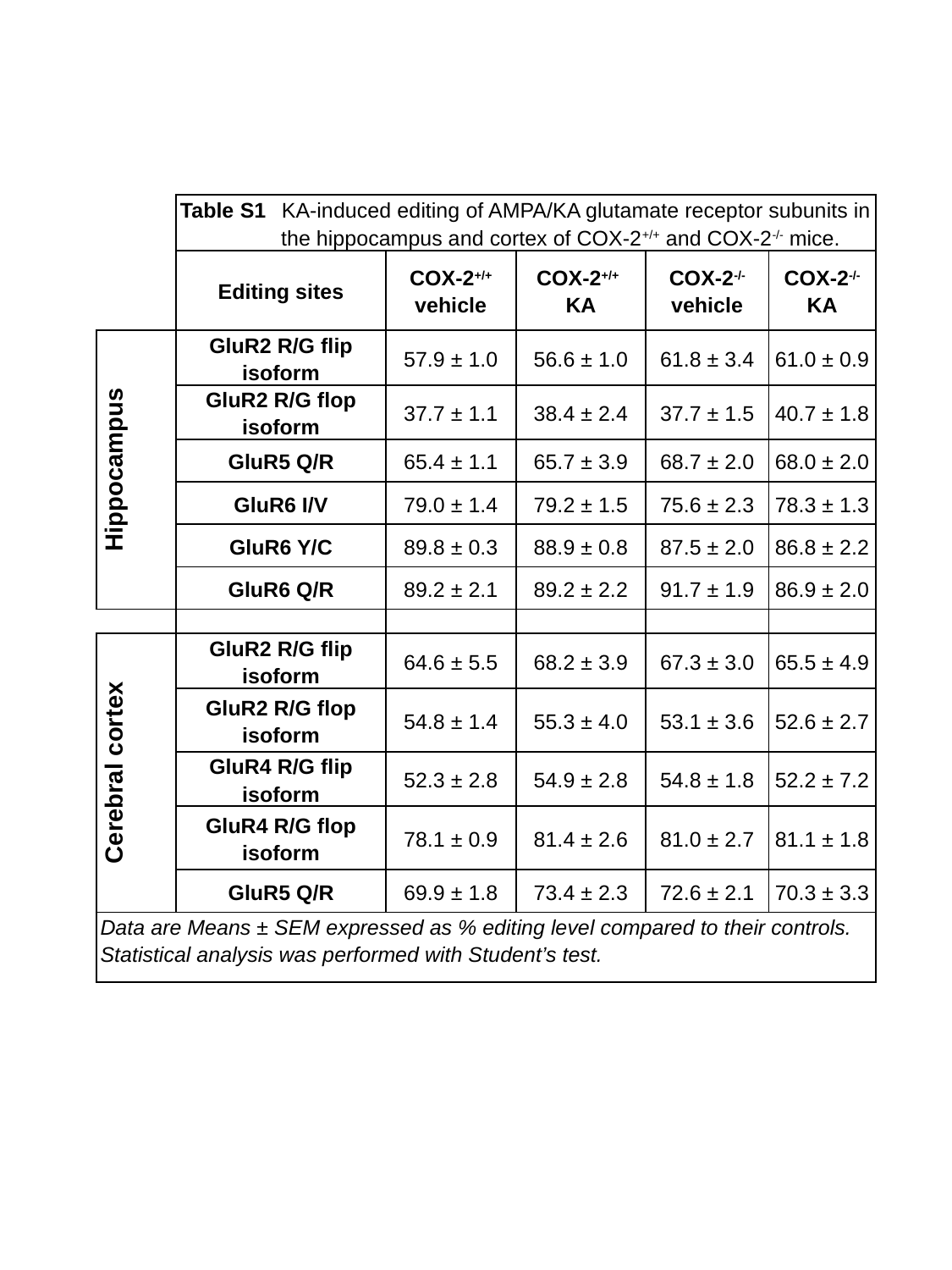

| | Table S1 KA-induced editing of AMPA/KA glutamate receptor subunits in the hippocampus and cortex of COX-2+/+ and COX-2-/- mice. | | | | |
| --- | --- | --- | --- | --- | --- |
| | Editing sites | COX-2+/+ vehicle | COX-2+/+ KA | COX-2-/- vehicle | COX-2-/- KA |
| Hippocampus | GluR2 R/G flip isoform | 57.9 ± 1.0 | 56.6 ± 1.0 | 61.8 ± 3.4 | 61.0 ± 0.9 |
| | GluR2 R/G flop isoform | 37.7 ± 1.1 | 38.4 ± 2.4 | 37.7 ± 1.5 | 40.7 ± 1.8 |
| | GluR5 Q/R | 65.4 ± 1.1 | 65.7 ± 3.9 | 68.7 ± 2.0 | 68.0 ± 2.0 |
| | GluR6 I/V | 79.0 ± 1.4 | 79.2 ± 1.5 | 75.6 ± 2.3 | 78.3 ± 1.3 |
| | GluR6 Y/C | 89.8 ± 0.3 | 88.9 ± 0.8 | 87.5 ± 2.0 | 86.8 ± 2.2 |
| | GluR6 Q/R | 89.2 ± 2.1 | 89.2 ± 2.2 | 91.7 ± 1.9 | 86.9 ± 2.0 |
| | | | | | |
| Cerebral cortex | GluR2 R/G flip isoform | 64.6 ± 5.5 | 68.2 ± 3.9 | 67.3 ± 3.0 | 65.5 ± 4.9 |
| | GluR2 R/G flop isoform | 54.8 ± 1.4 | 55.3 ± 4.0 | 53.1 ± 3.6 | 52.6 ± 2.7 |
| | GluR4 R/G flip isoform | 52.3 ± 2.8 | 54.9 ± 2.8 | 54.8 ± 1.8 | 52.2 ± 7.2 |
| | GluR4 R/G flop isoform | 78.1 ± 0.9 | 81.4 ± 2.6 | 81.0 ± 2.7 | 81.1 ± 1.8 |
| | GluR5 Q/R | 69.9 ± 1.8 | 73.4 ± 2.3 | 72.6 ± 2.1 | 70.3 ± 3.3 |
| Data are Means ± SEM expressed as % editing level compared to their controls. Statistical analysis was performed with Student’s test. | | | | | |
