# Supplementary material for: Altered mRNA Editing and Expression of Ionotropic Glutamate Receptors after Kainic Acid Exposure in Cyclooxygenase-2 Deficient Mice
Source: PLoS One. 2011 May 12;6(5):e19398. doi: 10.1371/journal.pone.0019398 (PMC3093380; doi:10.1371/journal.pone.0019398)
Supplement: Table S2 — KA-induced editing of AMPA/KA glutamate receptor subunits in hippocampus and cortex of wild type mice after pretreatment with celecoxib. Data are Means ± SEM expressed as % editing level compared to vehicle-injected wild type mice. Statistical analysis was performed with Student's test. (PPTX) [file pone.0019398.s002.pptx]

## Slide 1
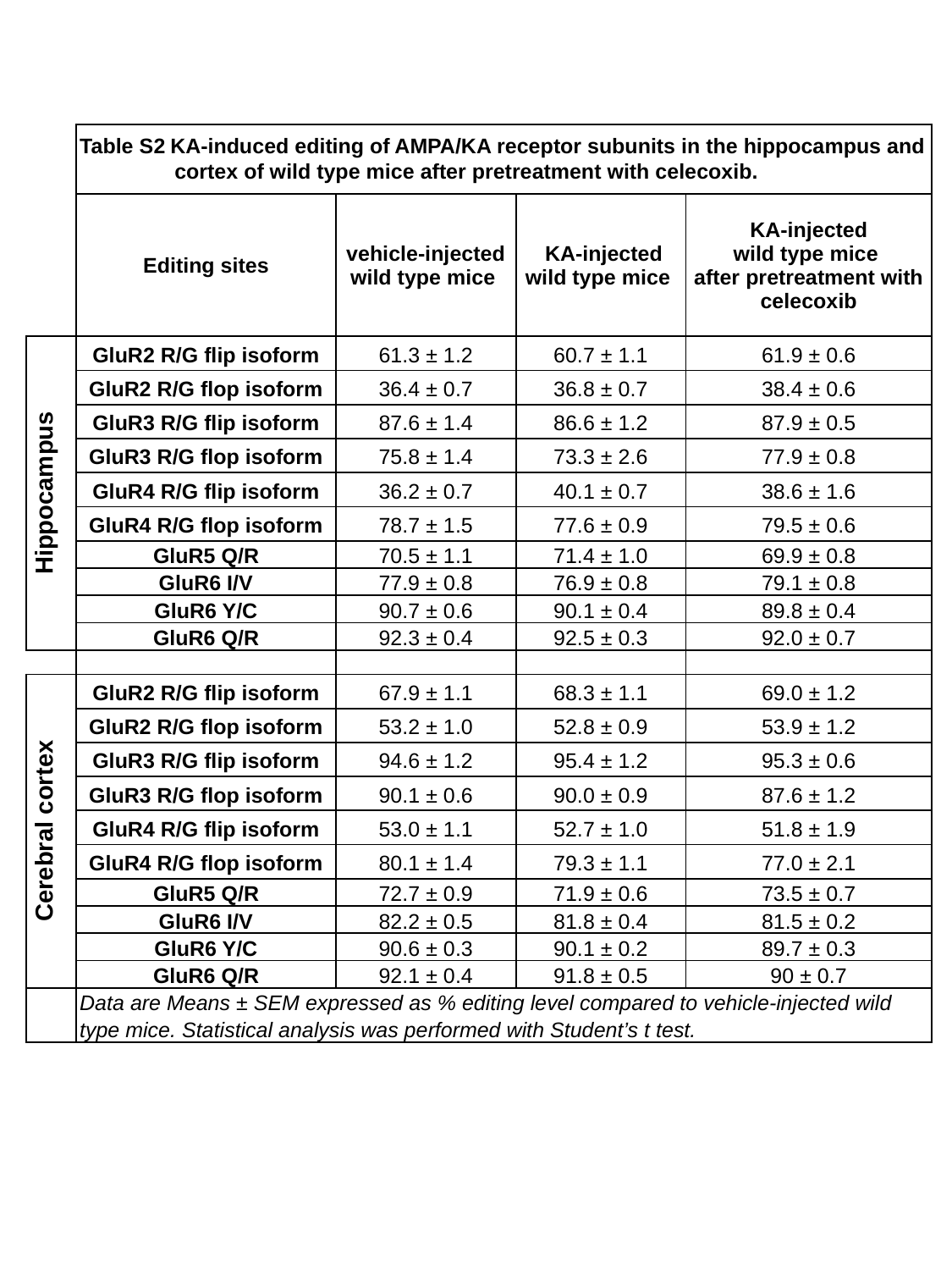

| | Table S2 KA-induced editing of AMPA/KA receptor subunits in the hippocampus and cortex of wild type mice after pretreatment with celecoxib. | | | |
| --- | --- | --- | --- | --- |
| | Editing sites | vehicle-injected wild type mice | KA-injected wild type mice | KA-injected wild type mice after pretreatment with celecoxib |
| Hippocampus | GluR2 R/G flip isoform | 61.3 ± 1.2 | 60.7 ± 1.1 | 61.9 ± 0.6 |
| | GluR2 R/G flop isoform | 36.4 ± 0.7 | 36.8 ± 0.7 | 38.4 ± 0.6 |
| | GluR3 R/G flip isoform | 87.6 ± 1.4 | 86.6 ± 1.2 | 87.9 ± 0.5 |
| | GluR3 R/G flop isoform | 75.8 ± 1.4 | 73.3 ± 2.6 | 77.9 ± 0.8 |
| | GluR4 R/G flip isoform | 36.2 ± 0.7 | 40.1 ± 0.7 | 38.6 ± 1.6 |
| | GluR4 R/G flop isoform | 78.7 ± 1.5 | 77.6 ± 0.9 | 79.5 ± 0.6 |
| | GluR5 Q/R | 70.5 ± 1.1 | 71.4 ± 1.0 | 69.9 ± 0.8 |
| | GluR6 I/V | 77.9 ± 0.8 | 76.9 ± 0.8 | 79.1 ± 0.8 |
| | GluR6 Y/C | 90.7 ± 0.6 | 90.1 ± 0.4 | 89.8 ± 0.4 |
| | GluR6 Q/R | 92.3 ± 0.4 | 92.5 ± 0.3 | 92.0 ± 0.7 |
| | | | | |
| Cerebral cortex | GluR2 R/G flip isoform | 67.9 ± 1.1 | 68.3 ± 1.1 | 69.0 ± 1.2 |
| | GluR2 R/G flop isoform | 53.2 ± 1.0 | 52.8 ± 0.9 | 53.9 ± 1.2 |
| | GluR3 R/G flip isoform | 94.6 ± 1.2 | 95.4 ± 1.2 | 95.3 ± 0.6 |
| | GluR3 R/G flop isoform | 90.1 ± 0.6 | 90.0 ± 0.9 | 87.6 ± 1.2 |
| | GluR4 R/G flip isoform | 53.0 ± 1.1 | 52.7 ± 1.0 | 51.8 ± 1.9 |
| | GluR4 R/G flop isoform | 80.1 ± 1.4 | 79.3 ± 1.1 | 77.0 ± 2.1 |
| | GluR5 Q/R | 72.7 ± 0.9 | 71.9 ± 0.6 | 73.5 ± 0.7 |
| | GluR6 I/V | 82.2 ± 0.5 | 81.8 ± 0.4 | 81.5 ± 0.2 |
| | GluR6 Y/C | 90.6 ± 0.3 | 90.1 ± 0.2 | 89.7 ± 0.3 |
| | GluR6 Q/R | 92.1 ± 0.4 | 91.8 ± 0.5 | 90 ± 0.7 |
| | Data are Means ± SEM expressed as % editing level compared to vehicle-injected wild type mice. Statistical analysis was performed with Student’s t test. | | | |
